# Assessing Thailand’s 1-3-7 surveillance strategy in accelerating malaria elimination

**DOI:** 10.1186/s12936-022-04229-z

**Published:** 2022-07-18

**Authors:** Prayuth Sudathip, Sathapana Naowarat, Suravadee Kitchakarn, Deyer Gopinath, Donal Bisanzio, Niparueradee Pinyajeerapat, David Sintasath, Jui A. Shah

**Affiliations:** 1 Division of Vector Borne Diseases, Department of Disease Control, Ministry of Public Health, Nonthaburi, Thailand; 2 Inform Asia: USAID’s Health Research Program, RTI International, Bangkok, Thailand; 3 World Health Organization, Nonthaburi, Thailand; 4 U.S. President’s Malaria Initiative, United States Agency for International Development (USAID), Regional Development Mission for Asia, Bangkok, Thailand

**Keywords:** Elimination, Surveillance, 1-3-7 Strategy

## Abstract

**Background:**

Thailand’s strong malaria elimination programme relies on effective implementation of its 1-3-7 surveillance strategy, which was endorsed and implemented nationwide in 2016. For each confirmed malaria patient, the Ministry of Public Health’s Division of Vector Borne Diseases (DVBD) ensures completion of case notification within 1 day, case investigation within 3 days, and foci investigation within 7 days. To date, there has not been a comprehensive assessment of the performance and achievements of the 1-3-7 surveillance strategy although such results could help Thailand’s future malaria elimination strategic planning.

**Methods:**

This study examined adherence to the 1-3-7 protocols, tracked progress against set targets, and examined geographic variations in implementation of the 1-3-7 strategy in the programme’s initial 5 years. An auto-regressive integrated moving average (ARIMA) time series analysis with seasonal decomposition assessed the plausible implementation effect of the 1-3-7 strategy on malaria incidence in the programme’s initial 5 years. The quantitative analysis included all confirmed malaria cases from public health and non-governmental community facilities from October 2014 to September 2021 (fiscal year [FY] 2015 to FY 2021) (n = 77,405). The spatial analysis included active foci with known geocoordinates that reported more than five cases from FY 2018 to FY 2021.

**Results:**

From FY 2017 to FY 2021, on-time case notification improved from 24.4% to 89.3%, case investigations from 58.0% to 96.5%, and foci investigations from 37.9% to 87.2%. Adherence to timeliness protocols did not show statistically significant variation by area risk classification. However, adherence to 1-3-7 protocols showed a marked spatial heterogeneity among active foci, and the ARIMA model showed a statistically significant acceleration in the reduction of malaria incidence. The 1-3-7 strategy national indicators and targets in Thailand have shown progressive success, and most targets were achieved for FY 2021.

**Conclusion:**

The results of Thailand’s 1-3-7 surveillance strategy are associated with a decreased incidence in the period following the adoption of the strategy although there is notable geographic variation. The DVBD will continue to implement and adapt the 1-3-7 strategy to accelerate progress toward malaria elimination. This assessment may be useful for domestic strategic planning and to other countries considering more intensive case and foci investigation and response strategies.

## Background

Thailand has a long history of conducting case investigations, case classifications, and focus investigation and response, as outlined in the Malaria Control Guidelines for Public Health Workers [1]. Between 2012 and 2015, the Division of Vector Borne Diseases (DVBD) in Thailand’s Ministry of Public Health (MOPH) reported significant reductions in the blood slide positivity rate to less than 5% among suspected cases with fever and in annual parasite incidence (API) to less than 1 per 1000 population (range 0.38–0.82) [2, 3]. Reaching these milestones allowed the DVBD to transition from a malaria control programme to a malaria elimination programme; details of this transition are presented in Lertpiriyasuwat et al. [4]. As part of the transition and based on results of a malaria programme review in 2015 [2, 3], the MOPH emphasized its commitment to surveillance as a core intervention by introducing the 1-3-7 malaria surveillance strategy in 2016.

The 1-3-7 malaria surveillance strategy is adapted from China, where it is considered a key factor in the country’s achievement of zero locally transmitted cases by 2017. The 1-3-7 strategy is operationalized with a simplified set of targets that outline responsibilities, actions, and the time frame for the crucial surveillance and response components of rapidly identifying infections and preventing them from spreading [2]. The time frame is delineated as follows: within 1 day of diagnosis (i.e., 24 h), local health staff are required to report the confirmed malaria case through the online Malaria Information System (MIS). Within 3 days, case investigation and classification must be completed to determine whether the case was locally acquired or imported. Finally, within 7 days, a focus investigation and tailored response based on the case investigation results and area stratification must be completed for each index case [5, 6]. Since the 1-3-7 strategy aims to reduce risk of onward transmission, response is similar for indigenous and imported cases except in areas without suitable vectors. Details on the implementation of the 1-3-7 strategy are documented in Lertpiriyasuwat et al. [4].

The 1-3-7 strategy builds on Thailand’s long-standing history of case notification, case classification, and focus investigation and response, with the addition of stricter time protocols to encourage adherence. Thailand’s National Malaria Elimination Strategy (NMES) 2017–2026 includes annual targets to support monitoring and evaluation of each component of the strategy. These targets were approved by the Cabinet of the Royal Thai Government in 2016 and aim to reach zero indigenous malaria cases by 2024 [2].

**Fig. 1:**
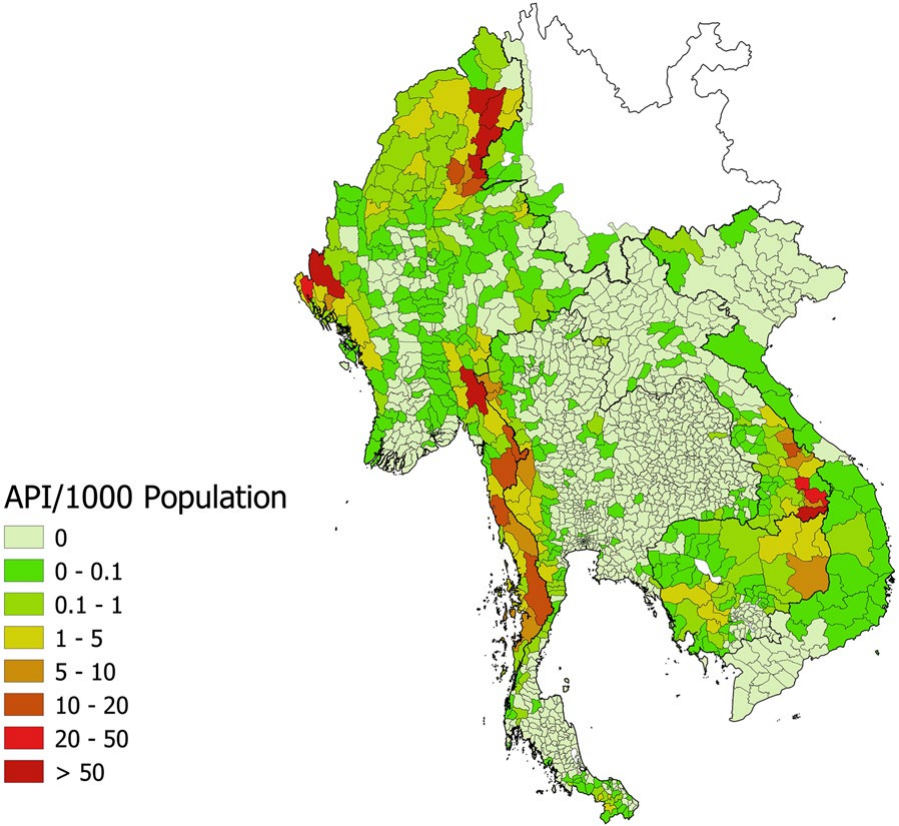
Malaria annual parasite incidence in GMS, FY 2021. Source: Malaria Elimination Database, Mekong Malaria Elimination Programme, 2021 (pers. commun.)

Since fiscal year (FY) 2015, the baseline year for the 1-3-7 strategy, malaria incidence in Thailand has declined from 0.37 per 1000 population to just 0.04 per 1000 population in FY 2021. The DVBD has also verified 42 of its 77 provinces as malaria-free, suggesting that the malaria elimination programme is progressing as intended. Remaining foci and high-transmission areas are concentrated in border provinces: Myanmar in the west, Cambodia in the east, and Malaysia in the South [7, 8]. The movement of people across international borders is a main challenge that continues to thwart malaria elimination in the Greater Mekong Subregion (GMS); districts bordering Thailand often have different strategies in place and, as seen in Fig. 1, may have higher malaria burden [9–12]. High population mobility and a large workforce of migrant workers make it difficult to track patients over time to ensure radical cure in this region of high *Plasmodium vivax* prevalence [6, 13–16]. Despite these challenges, 90.1% of *Plasmodium vivax* reportedly received radical cure and 81.5% of *Plasmodium falciparum* cases received single-dose primaquine in FY 2021, after steady improvement over time [17].

The DVBD has programmatically documented improvements in the timeliness of case reports and investigations [3, 4]. However, there has not been an in-depth examination of the performance and achievements of the 1-3-7 surveillance strategy in accelerating malaria elimination in Thailand. Adopting the 2024 elimination goal and related milestones without monitoring progress may lead to wasted resources and missed opportunities to enhance efficiency. This study examines adherence to the 1-3-7 protocols outlined in the Guidelines for Malaria Elimination for Public Health Workers, tracks progress against set targets in the NMES, and examines geographic variation in implementation and results [18]. This assessment of the strategy’s first 5 years of implementation will help Thailand’s future strategic planning and may be useful to other countries considering these more intensive case and foci investigation and response strategies.

## Methods

The analysis included all parasitologically confirmed cases (by microscopy or rapid diagnostic test) from public health and non-governmental community facilities reported from October 2014 to September 2021, representing FY 2015 to FY 2021. The study utilized FYs because Thailand’s malaria programme and database are based on FY targets. Quantitative malaria data were extracted from the national routine malaria information system, reviewed, and analyzed using IBM SPSS Statistics version 22. Spatial analyses were performed using R [19], Quantum GIS [20], and GeoDa [21]. To ensure high quality data and meaningful results, the authors verified, cross-checked, and cleaned data; the final dataset is described in the Results section. The analysis was threefold: (1) examining adherence to 1-3-7 protocols, (2) determining achievement of results compared to nationally set targets, and (3) understanding geographic variations that could explain continued transmission patterns.

In line with the National Strategic Operational Plan 2017–2021, FY 2015 was treated as a baseline year, and FY 2017 was treated as the first year of full-coverage implementation, leaving FY 2016, as a “buffer year” in measurement analyses [22]. The exclusion of FY 2016 accounted for the extraordinary effort that was required to develop, pilot test, and roll out materials and interventions to subnational officers to launch the 1-3-7 strategy. The buffer year also accommodated a policy change of stricter reactive case detection (RACD) inclusion criteria. Whereas RACD was previously conducted for 100 to 150 people (approximately 20 to 30 households) or a radius 1 to 2 km, in FY 2017 a narrower screening of 50 people (approximately 10 households) within 1–2 km was adopted nationally based on reducing malaria burden [18, 23]. Spatial analyses were conducted for FY 2018–FY 2021 only, when 1-3-7 performance was more consistent.

In Thailand, cases classified as “indigenous” refer to patients who contracted malaria in the village where they lived during the infection period [18, 24]. New cases refer to patients with confirmed malaria for the first time or at least 90 days subsequent to a previous malaria infection [18]. Due to the operational difficulty in differentiating *P. vivax* relapses from reinfections, these cases are usually reported as recurrences and later analysed in detail [17]; recurrences were treated as new cases for the purpose of this study. Any focus that has recorded an indigenous case in the previous 3 years (based on an annual focus classification cycle) is considered to be an “active focus” [25]. It is worth noting that the number of active foci has decreased dramatically, from 2227 in FY 2013 to 700 in FY 2019, as reported in Sudathip et al. [25, 26] and has further dropped to 469 in FY 2021. Adherence to the 1-3-7 requirements was measured with descriptive statistics on malaria case notification, case investigation and classification, foci investigation and classification, and responses results, as outlined in the National Strategic Operational Plan (Table 1). Results were consolidated by month from FY 2017–FY 2021.

**Table 1:** 1-3-7 strategy’s indicators and outputs and NMES indicators

	1-3-7 key and supplemental indicators
Day 1	Total malaria cases reported to the database
	Number and percentage of malaria cases reported to the malaria database within 1 day
Day 3	Number and percentage of malaria cases reported to the malaria database that were investigated
	Number and percentage of case investigations completed within 3 days
Day 7	Number of cases reported to the malaria database that require RACD screening
	Number and percentage of RACD events conducted
	Number and percentage of RACD events conducted within 7 days
	*Supplementary indicators without set targets*
	Number of individuals targeted for RACD screening
	Number and percentage of individuals screened during RACD
	Number and percentage of positive cases newly detected during RACD

	NMES outcome and impact indicators
	Percentage of districts with no indigenous malaria case transmission for at least 3 years
	Number of villages with indigenous malaria cases transmission

To assess achievement, values for key indicators were compared with national targets from the NMES. Values for supplementary indicators on RACD, for which the NMES does not specify annual targets (Table 1), were also reported to show progress over time. Outcome and impact indicators in the NMES track the number of villages and districts free from reported indigenous cases (Table 1).

To assess the plausible effect of the 1-3-7 strategy’s implementation on malaria incidence toward the elimination target of 2024, an auto regressive integrated moving average (ARIMA) was used to fit the time series model with trend and random components for active foci. Other key prevention and elimination strategies stayed the same over the study period, making this an appropriate approach to assess plausible results of the new strategy [26].

Thailand’s 1-3-7 protocol is intended to be standardized across all subnational units. However, it is possible that the strategy’s components are operationalized differently or that provincial teams face unique challenges, which could influence the strategy’s effect [24]. Spatial heterogeneity of adherence to 1-3-7 protocols was investigated among all active foci with known geocoordinates that reported more than five cases from FY 2018 to FY 2021.

The threshold of five cases was set arbitrarily to avoid inclusion of foci with very few investigated cases. Adherence was measured using the following four indicators: percentage of cases reported within 1 day, percentage of cases investigated within 3 days, percentage of cases for which RACD was completed within 7 days, and percentage of cases with the full 1-3-7 sequence performed without delays. A hot spot analysis based on the G* local spatial clustering test [27] was used to identify those areas with foci showing statistically significant lower adherence to the 1-3-7 schedules compared to all other foci. The significance of the results obtained by the G* local spatial test was computed by comparing observed values to a random case distribution (null hypothesis) by randomly re-assigning the values of the tested indicator across the foci. The statistical significance calculation was based on 10,000 Monte Carlo randomizations (*p* < 0.05, with Bonferroni correction).

## Results

Data from 77,405 malaria cases reported to the Malaria Information System (MIS) were split into 24,332 (31.4%) cases for FY 2015; 17,578 (22.7%) cases for the FY 2016 transition period; and 35,495 (45.9%) cases for the FY 2017–FY 2021 post-intervention period. Adherence and achievement analyses included all cases with complete data on 1-3-7 indicators, which were available for 15,887 (90.4%) of FY 2016 cases and 32,957 (92.9%) of FY 2017–FY 2021 post-intervention cases; the remainder were not included for analysis due to missing or duplicate data.

### Adherence to 1-3-7 protocols

#### Adherence to case notification within 1 day

**Fig. 2:**
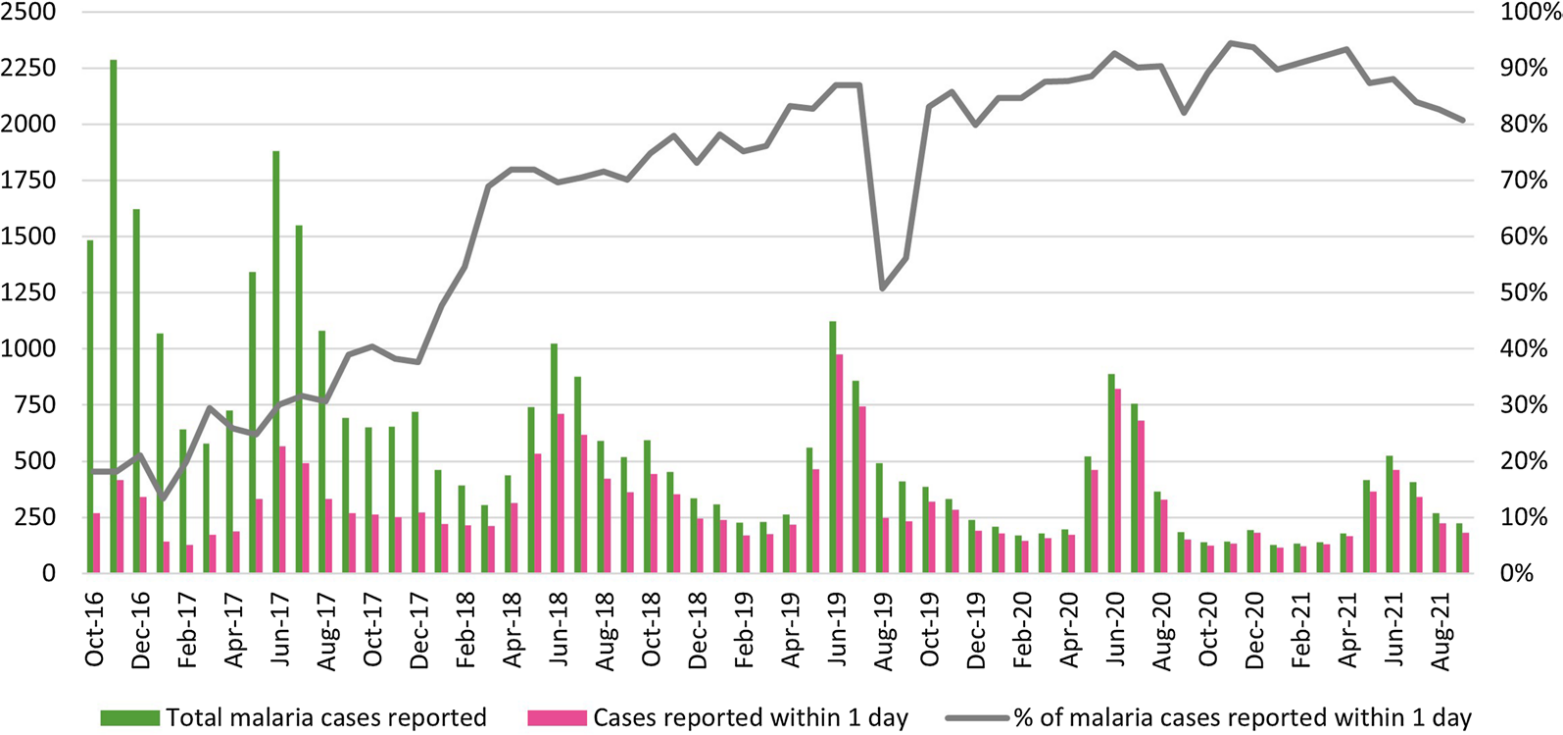
Number and percentage of malaria cases reported within 1 day, by month, FY 2017–FY 2021

The monthly proportion of confirmed malaria cases reported within 24 h to the system increased over the study period, from 18.2% in October 2016 to 80.7% in September 2021 (Fig. 2). This increase was steady despite the fact that reporting rates vary based on malaria seasonality. During the peak transmission month of June, the proportion of timely notification increased steadily from 30.1% in FY 2017 (567/1881) to 92.6% in FY 2020 (823/889) and 88.0% in FY 2021 (462/525). Note that there was a temporary drop of the timely notification proportion, to 56.2% at the end of FY 2019, due to an MIS database security breach in August 2019 that temporarily disabled data entry; malaria officers inputted data retrospectively 2 months later [28].

#### Adherence to case investigation within 3 days

**Fig. 3:**
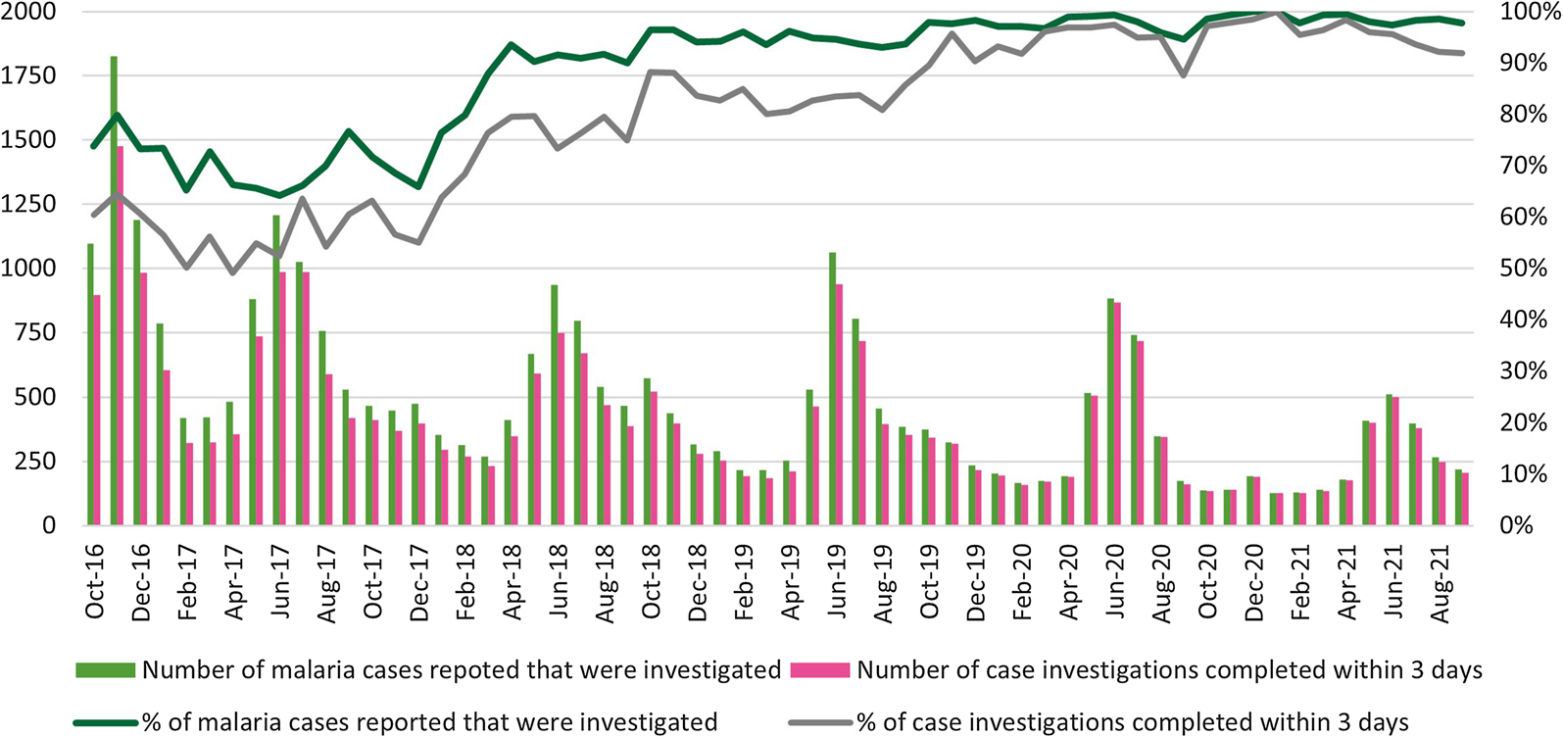
Number and percentage of malaria cases investigated within 3 days, by month, FY 2017–FY 2021

The monthly proportion of malaria cases investigated improved from 73.8% in October 2016 to 97.8% by September 2021, with substantial growth each year (Fig. 3). The proportion of case investigations within the 3-day requirement also improved over time, from 52.4% in June 2017 to 91.9% by September 2021, even after seasonality is considered. The proportion of malaria cases investigated during the peak season in June rose from 64.1% (1206/1881) in FY 2017 to 97.3% (511/525) in FY 2021. During the low transmission season, case investigation followed the same upward trend, rising from 72.7% (421/579) in FY 2017 to 99.3% (139/140) in FY 2021. Reporting timeliness also increased from 52.4% and 56.1% in the FY 2017 peak and low seasons, respectively, to 95.6% and 96.4% in the FY 2021 peak and low seasons, respectively.

#### Adherence to foci investigation and response within 7 days

**Fig. 4:**
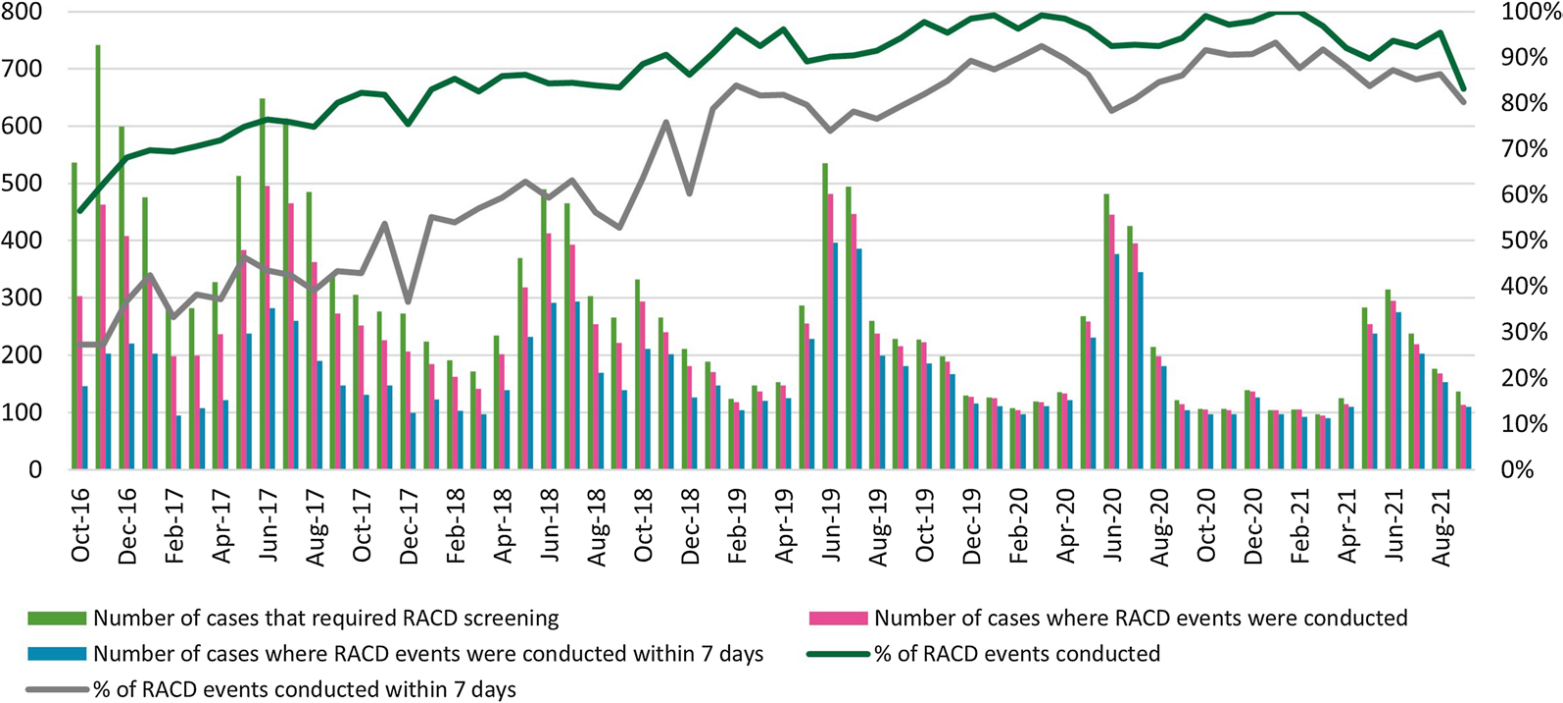
Number and percentage of foci investigated and responded to within 7 days, FY 2017–FY 2021

The monthly proportion of RACD conducted among the cases that required RACD improved substantially over the study period, from 56.5% in October 2016 to 83.2% by September 2021 (Fig. 4). The proportion of RACD events conducted on time (within 7 days of case notification) also made dramatic improvement over the study period, from 48.2% to 80.3%. These proportions did not vary much between high and low transmission seasons.

#### Comparison by area classification

Comparative t-tests showed that adherence to the DVBD’s timeliness protocols did not show statistically significant variation by area classification. Before and during the buffer year of 1-3-7 implementation, timely case reporting was higher among active foci than among cleared foci with an index case (*p* < 0.05). Although active foci continued to show more timely surveillance and response than cleared foci, in the post-intervention period, these differences were not statistically significant.

### Achievements of the 1-3-7 surveillance strategy compared to national targets

The second part of this analysis compared actual results to targets laid out by FY, divided into output results and outcome/impact results (see Table 2 for summarized results). The NMES uses FY 2015 as a baseline year.

**Table 2:** Achievement of 1-3-7 strategy, national indicators, and targets for FY 2017–FY 2020

Indicators		FY 2015	FY 2017	FY 2018	FY 2019	FY 2020	FY 2021
Output indicators							
Percentage of malaria cases reported in the MIS within 24 h	**Target**		**50%**	**60%**	**70%**	**80%**	**90%**
	**Achievement**	**18.0%**	**24.4%**	**59.6%**	**77.1%**	**87.8%**	**89.3%**
Total number of malaria cases reported to the database		24,332	14,954	7368	5845	4421	2835
Number of malaria cases reported to the malaria database within 1 day		4383	3643	4388	4508	3881	2531
Percentage of malaria cases investigated within 3 days	**Target**		**85%**	**90%**	**95%**	**95%**	**95%**
	**Achievement**	**44.1%**	**58.0%**	**70.4%**	**84.0%**	**94.7%**	**96.5%**
Number of malaria cases reported to the malaria database		24,332	14,954	7368	5845	4421	2,835
Number of malaria cases reported to the malaria database that were investigated		15,992	10,614	6140	5532	4331	2785
Number of case investigations completed within 3 days		10,742	8674	5185	4907	4185	2735
Percentage of malaria cases investigated with foci response conducted within 7 days	**Target**		**30%**	**50%**	**60%**	**70%**	**80%**
	**Achievement**	**35.7%**	**37.9%**	**55.2%**	**75.3%**	**84.1%**	**87.2%**
Number of cases reported to the malaria database that require RACD screening		7704	5848	3566	3225	2557	1936
Number of RACD events conducted		5410	4119	2973	2928	2433	1824
Number of RACD events conducted within 7 days		2750	2214	1969	2428	2150	1689
Outcome and impact indicators							
Number and percentage of districts with no indigenous malaria case transmission for at least 3 years (N = 928)	**Target**		**696 (75%)**	**743 (80%)**	**789 (85%)**	**836 (90%)**	**882 (95%)**
	**Achievement**	**632**	**748 (80%)**	**767 (83%)**	**788 (85%)**	**798 (86%)**	**813 (88%)**
Number of villages with indigenous malaria case transmission	**Target**		**3610**	**2707**	**1895**	**1232**	**739**
	**Achievement**	**5552**	**2310**	**1676**	**1382**	**1187**	**469**

#### Output indicators

Overall, results on output indicators were strong. First, malaria cases reported in the MIS within the 1-day (or 24-h) period showed consistent improvement over time, with rates reaching targets in FY 2019, FY 2020, and FY 2021 (77.1%, 87.8%, and 89.3%, respectively). Second, the proportion of cases investigated within 3 days dramatically increased from baseline to 96.5%, exceeding the FY 2021 target (95%). Lastly, for foci investigation and response conducted within 7 days, the outputs met the targets in every FY, with the FY 2020 and FY 2021 proportions of 84.1% and 87.2% both surpassing the FY 2021 target.

The authors also examined the supplemental output indicators for which targets were not defined in the NMES, with results summarized in Table 3. During FY 2017–FY 2021, 14,277 index cases triggered RACD (83.3% of total cases eligible for RACD), leading to 866,920 people being screened: 1513 (0.17%) were positive for malaria infection. Each year, the number of individuals screened during RACD events dropped, with the proportional yield of positive cases also decreasing from 0.23% (507 cases) in FY 2017 to 0.11% (133 cases) in FY 2021. RACD methods produced nearly equal yields for both active foci and cleared foci with a confirmed index case (0.19% versus 0.16%).

**Table 3:** Performance of RACD events conducted among foci investigations for FY 2017–FY 2021

Indicator	Achievement				
	2017	2018	2019	2020	2021
Number of individuals screened during RACD	222,991	182,275	176,162	159,892	125,600
Number of positive cases newly identified using RACD	507	311	314	248	133
Percent found positive from RACD events	0.23%	0.17%	0.18%	0.16%	0.11%

#### Outcome/impact indicators

For the outcome indicator of percentage of districts without local transmission for at least 3 years (among a total of 928 districts in Thailand), targets were reached every FY between 2017 and 2019 (80%, 83%, and 85% respectively). Progress plateaued in FY 2020 and FY 2021, whereby district achievement reached 86% and 85%, missing the 90% and 95% targets, respectively. The second outcome indicator is the number of villages with malaria transmission, which dropped from 2310 in FY 2017 to 469 in FY 2021 (Table 2). This indicator has shown better-than-expected results, with substantial reductions beyond the set target each year, showing strides in interrupting community transmission.

**Fig. 5:**
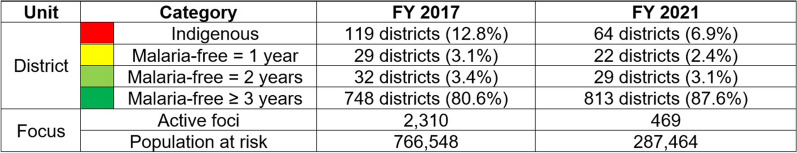
District-level improvements in malaria free status from FY 2017 and FY 2021. Source: DVBD, MOPH, 2021 (pers. commun.)

These results were supplemented by other data collected by the DVBD on the population at risk in active foci, which from FY 2017 to FY 2021 dropped from 766,548 to 287,464 (Fig. 5), and a decrease in the number of active foci from 2310 to 469 for the same period. Because the DVBD’s unit of analysis for daily assessments of routine data was the focus level, the programme’s MIS collated more granular geotemporal data on malaria-free area classification than what was required for NMES reporting.

#### Observed and predicted trend in incidence

**Fig. 6:**
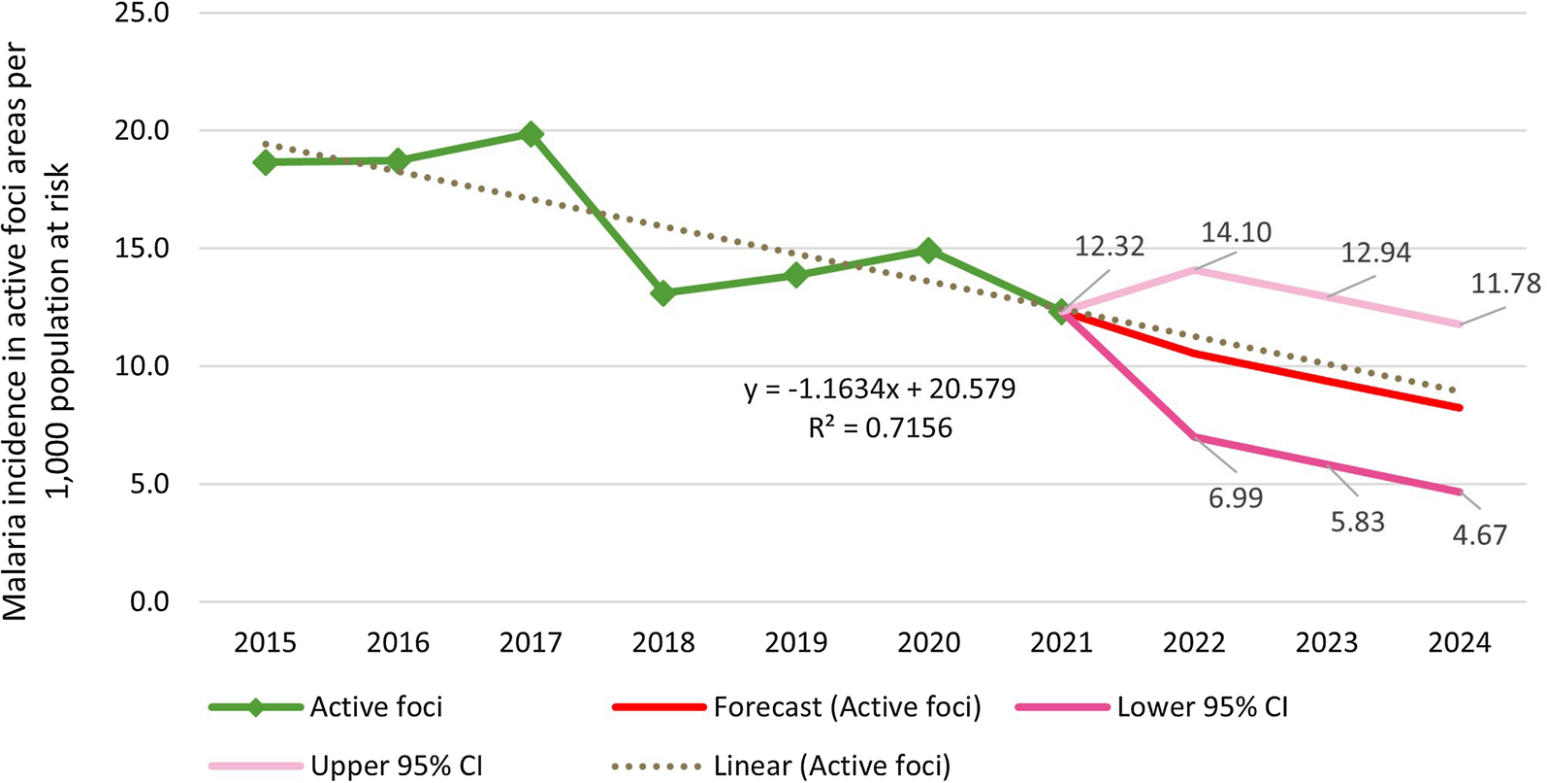
Observed and forecast malaria incidence per 1000 population at risk in active foci areas FY 2015–FY 2024

The annual positive rates of all malaria species were used to establish an ARIMA model in active foci areas (R2 = 0.72) (Fig. 6). Prior to the 1-3-7 strategy’s launch, malaria incidence among active foci was decreasing annually by 1.02 per 1000 population at risk. After the launch in FY 2016, the ARIMA model showed an additional reduction to 1.31 cases per 1000 population at risk per year. Each subsequent year has seen further reductions, averaging 1.36 (*p* = 0.62) annually, indicating an acceleration in the reduction of malaria incidence during the implementation period. The trend is forecasted to continue through FY 2024.

### Spatial analyses among active foci

**Fig. 7:**
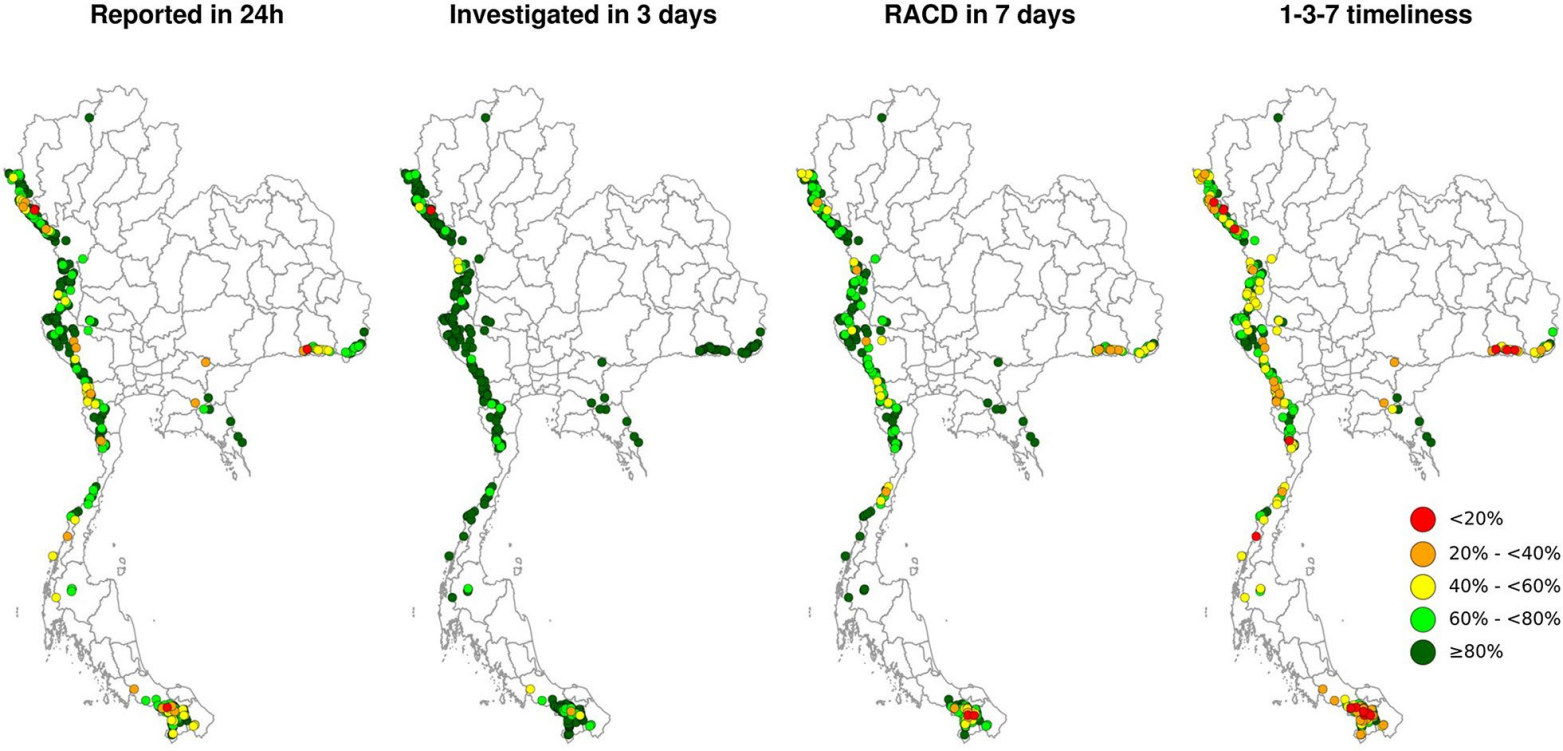
Spatial pattern of adherence to 1-3-7 protocols among active foci in Thailand, FY 2018–FY 2021

**Fig. 8:**
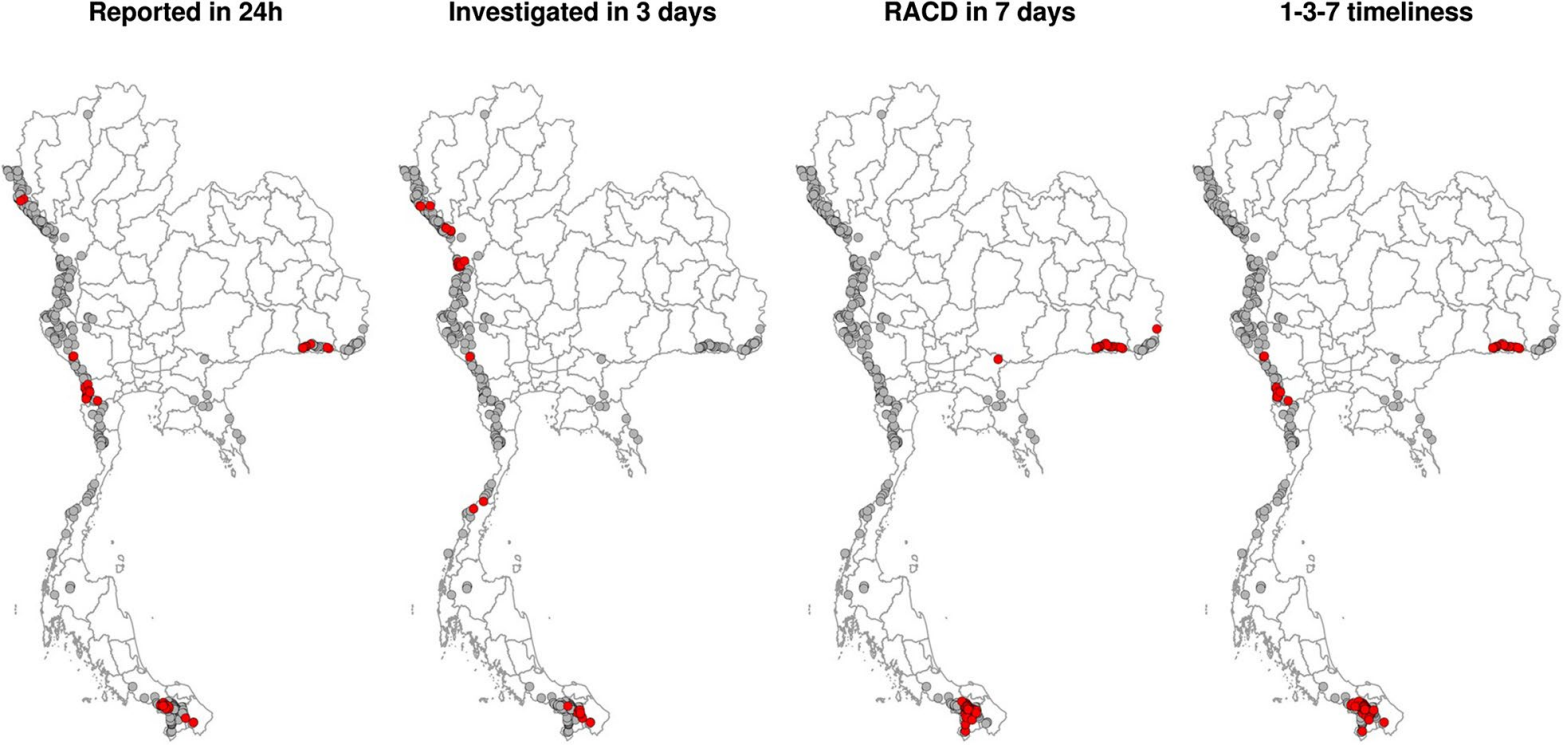
Active foci with significantly lower adherence to 1-3-7 protocols, FY 2018–FY 2021 The red dots indicate significant hot spots (p < 0.05) of low adherence to 1-3-7 timing identified by the G* local spatial test

During the study period, active foci were clustered at Thailand’s international borders. Adherence to 1–3-7 protocols showed a marked spatial heterogeneity among active foci (Fig. 7), with southern and eastern foci reporting lowest adherence. Among the indicators examined, timely case investigation showed the highest results and lowest spatial heterogeneity (Fig. 7); however, some foci in high-burden areas in the northwest showed poor adherence (*p* < 0.05). The G* test identified foci with significantly (*p* < 0.05) lower adherence to overall 1-3-7 timeliness, mostly in Sisaket province in the east, Kanchanaburi and Ratchaburi provinces in the west, and Yala province in the south (Fig. 8).

## Discussion

The 1-3-7 strategy is an innovative surveillance and response intervention for accelerating progress toward Thailand’s malaria elimination goal by 2024. Even if only one malaria case is detected, local health personnel must respond immediately per standard operating procedures, making the strategy quite sensitive to epidemiological changes [3, 4]. Adherence rates show that the protocols are being successfully implemented, with case notifications within 1 day increasing from 24.4% to 89.3%, case investigations within 3 days increasing from 58.0% to 96.5%, and foci investigations and response within 7 days increasing from 37.9% to 87.2% during the first 5 years of implementation.

Data quality is high, particularly in terms of completeness and timeliness; in fact, the DVBD’s coordination and communication across subnational units has been cited as a success factor for the 1-3-7 strategy [3, 4]. The national team provides close operational support to subnational teams on data quality and compliance with 1-3-7 protocols. The DVBD regularly monitors for unusual data, such as an increase in cases compared to historic data or a case identified in a province already verified as malaria free. The programme immediately and directly communicates queries to specific subnational officers via its malaria group mobile chat application with more than 400 malaria members from across the country from every level of the health system. Subnational officers respond publicly, describing investigation efforts, providing contextual information and photographs, and following up to share subsequent results or requesting further guidance. The DVBD also distributes a monthly report collating unusual events and data trends to all provincial offices, in addition to a monthly report tracking progress on national indicators to leadership in the Department of Disease Control.

To ensure continued progress toward the ultimate goal of zero malaria, the DVBD is conducting further analyses on each component of the 1-3-7 strategy to better understand reasons behind the documented geographic variation. Thailand’s routine surveillance data show that high-incidence provinces have many more active foci per province and more cases per focus. However, the provinces with low adherence identified by the spatial analysis have unique challenges related to drug efficacy (Sisaket in the east), migration patterns (Kanchanaburi and Ratchaburi in the west), and civic unrest (Yala in the south). Assessing areas of persistent transmission can help target resources to accelerate elimination, so the DVBD is currently developing a comprehensive statistical model that includes 1-3-7 implementation, environmental factors, and social and behavioral characteristics.

The 1-3-7 strategy national indicators and targets in Thailand have shown progressive success—more than 80% achievement in every component. Most targets were achieved for FY 2021, suggesting that the DVBD set out ambitious but realistic goals in the NMES. Because few other changes were implemented in the malaria control programme at the time of the 1-3-7 adoption, the results of this study suggest it is plausible that the strategy is associated with the documented 80% decline in malaria burden from FY 2017 to FY 2021 and the notable increase in malaria-free foci and districts.

Of course, the context also evolves, particularly as the protracted COVID-19 pandemic poses a serious additional challenge to global progress toward malaria elimination [29]. In January 2020, Thailand became the second country in the world to confirm a COVID-19 case. Several protective movement restrictions were implemented in the subsequent months, resulting in just 3553 cases of COVID-19 recorded in FY 2020 [30]. These cases were mostly limited to urban areas, contrary to malaria cases that are concentrated in forested border areas, so there was limited overlap in epidemiology. However, in FY 2021, over 1.6 million cases were reported as the epidemiology widened to affect most areas of the country [30]. COVID-19 cases peaked during the high malaria transmission period from July to September 2021 and may have affected malaria interventions by overwhelming health systems, altering population movement and health seeking behaviors, and diverging resources and frontline workers. As seen in this study’s results, 1-3-7 adherence rates were higher in FY 2020 than FY 2021, particularly for focus investigation and response, thereby meriting further analyses. Overall, Thailand has maintained its results for 1-3-7 indicators despite COVID-19, likely due to high-level support and the presence of a strong vertical programme complemented by a wide community health worker network.

Thailand’s declining malaria burden supports its collaborative regional goals and programmes. Since 2000, GMS countries have successfully reduced the reported number of all malaria species cases by 90% and *P. falciparum* cases by 97% [29]. This is coupled with a decline in malaria deaths from 6000 in 2000 to 10 in 2020 [31] in the GMS. Altogether, this is remarkable progress toward the region’s collective goal of malaria elimination by 2030. Following China and Thailand, Myanmar, Cambodia, and Lao PDR began piloting the 1-3-7 strategy [32, 33]. Continuation of the close partnership among GMS countries will help support regional goals and address some of the most challenging border hot spots identified by the spatial analysis. There is also historical success of cross-border and civilian-military cooperation in Sisaket province [34]. A starting point for expanded programming could be cross-border notification and foci response, utilizing the networks of community health workers that are acutely familiar with local populations, and providing additional training to boost 1-3-7 adherence in those areas.

### Limitations

This assessment is not a true impact evaluation of the malaria elimination programme or the 1-3-7 surveillance intervention of Thailand. There is substantial guidance on conducting robust impact evaluations for full-scale malaria interventions; however, these models have been designed for contexts with substantial malaria burden [33, 35, 36]. Even the guidance issued for lower transmission settings is designed for API that is higher than those in Thailand [37]. The ARIMA model is based on case burden and is a useful method to assess the 1-3-7 strategy’s plausible effect on incidence. However, the model does not account for important programmatic and contextual factors.

This study used robust routine data from Thailand’s national MIS [36]. Data completeness, as reported in the results section, is less than 100%, which is normal for routine data but requires appropriate interpretation. The cases not captured in the MIS are likely to be patients that have a deliberate reason to avoid the surveillance system or the formal public health system, and it is difficult to assess how many cases may be missed. Furthermore, response data are particularly challenging to interpret. RACD results are reported in aggregate at the foci level so coverage up to the required 50 individuals, 10 households, or 2 km is difficult to ascertain, as are case-level geolocation or demographics. The DVBD and its partners are working to ensure that the MIS is a complete and comprehensive source of malaria data before the 2024 elimination target.

### Next steps and road to elimination

Although the 1-3-7 strategy has been successfully implemented and results have shown steady improvements, efforts must be sustained to address emerging challenges due to COVID-19, migration patterns, or sociopolitical dynamics in the GMS [7, 16, 38]. Preventing resurgence and reintroduction of malaria are also growing priorities for the DVBD, based on lessons learned elsewhere [39, 40]. These investments in malaria surveillance can enhance Thailand’s health security and preparedness in crucial areas such as rapid detection, alert, and response, thereby building resilience against future infectious disease threats.

The DVBD has been intensifying its strategies to ensure appropriate surveillance for very low-transmission settings [41, 42]. Such strategies include enhancements to foci response and active surveillance protocols, advocacy for policy legislation, resources for additional staff trainings on accurate case management, support for improved data recording and reporting, and involvement of the private sector and military to ensure that the surveillance system is capturing all cases [34, 43]. Effectively reaching marginalized populations may require innovative solutions, particularly as malaria burden and risk perception falls [5, 44].

## Conclusions

The encouraging results of Thailand’s 1-3-7 surveillance strategy are in line with the dramatically decreased incidence seen in the time period following the strategy’s adoption. The malaria programme will continue its emphasis on implementing and applying Thailand’s 1-3-7 strategy, perhaps with additionally tailored activities for varying zones of transmission or that capitalize on community health networks and cross-border collaboration. The 1-3-7 strategy supports the DVBD’s goals to achieve and sustain malaria elimination and could be a useful example for other countries in the GMS aiming to eliminate malaria.

## Data Availability

The visualizations supporting the conclusions of this article are available in the Malaria Online repository, http://malaria.ddc.moph.go.th/.

## Abbreviations

ARIMA: Auto-regressive integrated moving average
COVID-19: Coronavirus disease 2019
DVBD: Division of Vector Borne Diseases
FY: Fiscal year
GMS: Greater Mekong Subregion
MIS: Malaria Information System
MOPH: Ministry of Public Health
NMES: National Malaria Elimination Strategy
PMI: President’s Malaria Initiative
RACD: Reactive case detection
USAID: United States Agency for International Development
